# 3-(4-Chloro­phen­yl)-1-(2-methyl-4-phenyl­quinolin-3-yl)prop-2-en-1-one

**DOI:** 10.1107/S1600536811004661

**Published:** 2011-02-12

**Authors:** R. Prasath, P. Bhavana, Ray J. Butcher, Jerry P. Jasinski

**Affiliations:** a Chemistry Group, BITS, Pilani, K. K. Birla Goa Campus, Goa 403 726, India; b Department of Chemistry, Howard University, 525 College Street NW, Washington, DC 20059, USA; c Department of Chemistry, Keene State College, 229 Main Street, Keene, NH 03435-2001, USA

## Abstract

The crystal structure of the title compound, C_25_H_18_ClNO, shows that the mol­ecules are isolated and not involved in inter­molecular C—H⋯O or C—H⋯Cl inter­actions. However, the phenyl and quinoline rings are involved in π–π inter­actions [centroid–centroid distance = 3.8829 (9) Å].

## Related literature

For background details and the biological activity of quinolines, see: Markees *et al.* (1970 ▶); Campbell *et al.* (1998 ▶); Bhat *et al.* (2005 ▶). For the biological activity of chalcones, see: Wu *et al.* (2006 ▶).
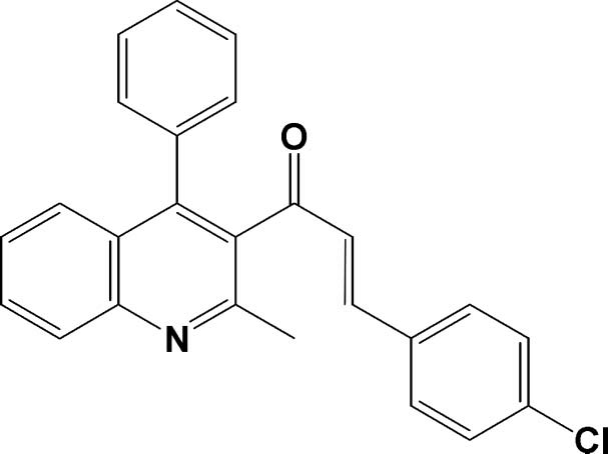


## Experimental

### 

#### Crystal data

C_25_H_18_ClNO
*M* *_r_* = 383.85
Triclinic, 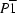

*a* = 6.5376 (2) Å
*b* = 10.0345 (4) Å
*c* = 15.6545 (6) Å
α = 90.845 (3)°
β = 95.521 (3)°
γ = 107.035 (3)°
*V* = 976.36 (6) Å^3^
*Z* = 2
Cu *K*α radiation
μ = 1.84 mm^−1^
*T* = 295 K
0.52 × 0.18 × 0.12 mm

#### Data collection

Oxford Diffraction Xcalibur Ruby Gemini diffractometer
Absorption correction: multi-scan (*CrysAlis PRO*; Oxford Diffraction, 2009 ▶) *T*_min_ = 0.544, *T*_max_ = 1.000
7607 measured reflections
4065 independent reflections
3402 reflections with *I* > 2σ(*I*)
*R*_int_ = 0.019

#### Refinement

*R*[*F* ^2^ > 2σ(*F* ^2^)] = 0.049
*wR*(*F* ^2^) = 0.160
*S* = 1.04
4065 reflections
254 parameters
H-atom parameters constrained
Δρ_max_ = 0.29 e Å^−3^
Δρ_min_ = −0.24 e Å^−3^

### 

Data collection: *CrysAlis PRO* (Oxford Diffraction, 2009 ▶); cell refinement: *CrysAlis PRO*; data reduction: *CrysAlis PRO*; program(s) used to solve structure: *SHELXS97* (Sheldrick, 2008 ▶); program(s) used to refine structure: *SHELXL97* (Sheldrick, 2008 ▶); molecular graphics: *SHELXTL* (Sheldrick, 2008 ▶); software used to prepare material for publication: *SHELXTL*.

## Supplementary Material

Crystal structure: contains datablocks I, global. DOI: 10.1107/S1600536811004661/ez2231sup1.cif

Structure factors: contains datablocks I. DOI: 10.1107/S1600536811004661/ez2231Isup2.hkl

Additional supplementary materials:  crystallographic information; 3D view; checkCIF report

## Figures and Tables

**Fig. 1.**
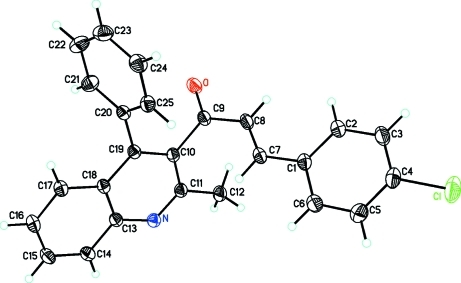
Diagram of the title compound, C25H18ClNO, showing atom labeling.

**Fig. 2.**
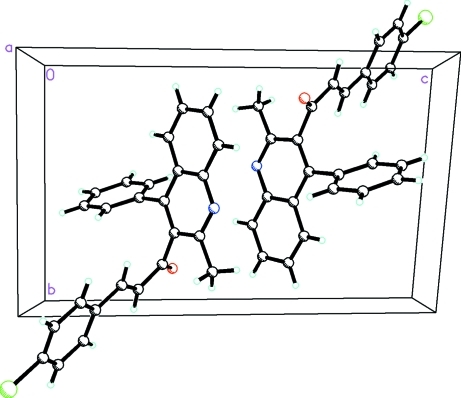
The molecular packing for C25H18ClNO viewed down the a axis.

## supplementary crystallographic information

### Comment

Quinoline derivatives are very important compounds because of their wide occurrence in natural products and biologically active compounds (Markees *et al.*, 1970; Campbell *et al.*, 1998). Additionally, chalcone derivatives are attracting the increasing interest of many researchers, because of their bioactivity such as antimicrobial, antimalarial, anticancer, antiviral, antitumor activities (Bhat *et al.*, 2005). Introduction of the quinoline scaffold into chalcone compounds can bring about significant changes in biological effects (Wu *et al.*, 2006).

The crystal structure of the title compound shows that the molecules are isolated and not involved in intermolecular interactions. However, both the phenyl ring and the quinoline rings are involved in π–π interactions (centroid to centroid distances of 3.428 (2) and 3.770 (2) Å, respectively).

### Experimental

A mixture of 3-acetyl-2-methyl-4-phenylquinoline (2.61 g, 0.01 M), 4-chlorobenzaldehyde (1.40 g, 0.01 M) and KOH (1.12 g, 0.02 M ) in distilled ethanol (20 ml) was stirred for 12 h at room temperature. The resulting mixture was neutralized with dilute acetic acid. The resultant solid was filtered, dried and purified by column chromatography using 1:1 mixture of ethyl acetate and hexane. Re-crystallization was by slow evaporation of acetone solution of (I) which yielded colourless needle type crystals. M.pt. 453-455 K. Yield: 72%.

### Refinement

H atoms were placed in geometrically idealized positions and constrained to ride on their parent atoms with a C—H distances of 0.93 and 0.96 Å *U*_iso_(H) = 1.2*U*_eq_(C) and 0.98 Å for CH_3_ [*U*_iso_(H) = 1.5*U*_eq_(C)].

### Crystal data

**Table:**

C_25_H_18_ClNO	*Z* = 2
*M**_r_* = 383.85	*F*(000) = 400
Triclinic, *P*1	*D*_x_ = 1.306 Mg m^−^^3^
Hall symbol: -P 1	Cu *K*α radiation, λ = 1.54184 Å
*a* = 6.5376 (2) Å	Cell parameters from 4929 reflections
*b* = 10.0345 (4) Å	θ = 5.3–77.1°
*c* = 15.6545 (6) Å	µ = 1.84 mm^−^^1^
α = 90.845 (3)°	*T* = 295 K
β = 95.521 (3)°	Needle, colorless
γ = 107.035 (3)°	0.52 × 0.18 × 0.12 mm
*V* = 976.36 (6) Å^3^	

### Data collection

**Table:**

Oxford Diffraction Xcalibur Ruby Gemini diffractometer	4065 independent reflections
Radiation source: Enhance (Cu) X-ray Source	3402 reflections with *I* > 2σ(*I*)
graphite	*R*_int_ = 0.019
Detector resolution: 10.5081 pixels mm^-1^	θ_max_ = 77.4°, θ_min_ = 5.3°
ω scans	*h* = −3→8
Absorption correction: multi-scan (*CrysAlis PRO*; Oxford Diffraction, 2009)	*k* = −12→12
*T*_min_ = 0.544, *T*_max_ = 1.000	*l* = −19→19
7607 measured reflections	

### Refinement

**Table:**

Refinement on *F*^2^	Primary atom site location: structure-invariant direct methods
Least-squares matrix: full	Secondary atom site location: difference Fourier map
*R*[*F*^2^ > 2σ(*F*^2^)] = 0.049	Hydrogen site location: inferred from neighbouring sites
*wR*(*F*^2^) = 0.160	H-atom parameters constrained
*S* = 1.04	*w* = 1/[σ^2^(*F*_o_^2^) + (0.1125*P*)^2^ + 0.0628*P*] where *P* = (*F*_o_^2^ + 2*F*_c_^2^)/3
4065 reflections	(Δ/σ)_max_ = 0.001
254 parameters	Δρ_max_ = 0.29 e Å^−^^3^
0 restraints	Δρ_min_ = −0.24 e Å^−^^3^

### Special details

**Table:**

Geometry. All e.s.d.'s (except the e.s.d. in the dihedral angle between two l.s. planes) are estimated using the full covariance matrix. The cell e.s.d.'s are taken into account individually in the estimation of e.s.d.'s in distances, angles and torsion angles; correlations between e.s.d.'s in cell parameters are only used when they are defined by crystal symmetry. An approximate (isotropic) treatment of cell e.s.d.'s is used for estimating e.s.d.'s involving l.s. planes.
Refinement. Refinement of *F*^2^ against ALL reflections. The weighted *R*-factor *wR* and goodness of fit *S* are based on *F*^2^, conventional *R*-factors *R* are based on *F*, with *F* set to zero for negative *F*^2^. The threshold expression of *F*^2^ > σ(*F*^2^) is used only for calculating *R*-factors(gt) *etc*. and is not relevant to the choice of reflections for refinement. *R*-factors based on *F*^2^ are statistically about twice as large as those based on *F*, and *R*- factors based on ALL data will be even larger.

### Fractional atomic coordinates and isotropic or equivalent isotropic displacement parameters (Å^2^)

**Table:**

	*x*	*y*	*z*	*U*_iso_*/*U*_eq_	
Cl	−0.41045 (10)	−0.24622 (7)	0.99317 (4)	0.1004 (3)	
O	0.6524 (2)	0.15628 (12)	0.65464 (9)	0.0678 (3)	
N	0.22876 (19)	0.41447 (12)	0.55076 (8)	0.0485 (3)	
C1	0.0479 (2)	0.02060 (15)	0.82219 (9)	0.0505 (3)	
C2	0.0750 (3)	−0.10510 (17)	0.85056 (12)	0.0603 (4)	
H2A	0.1889	−0.1338	0.8340	0.072*	
C3	−0.0651 (3)	−0.18756 (18)	0.90290 (12)	0.0671 (4)	
H3A	−0.0465	−0.2714	0.9212	0.081*	
C4	−0.2326 (3)	−0.14364 (19)	0.92754 (11)	0.0638 (4)	
C5	−0.2633 (3)	−0.0196 (2)	0.90095 (13)	0.0696 (4)	
H5A	−0.3765	0.0092	0.9181	0.083*	
C6	−0.1218 (3)	0.06090 (18)	0.84822 (12)	0.0620 (4)	
H6A	−0.1416	0.1444	0.8299	0.074*	
C7	0.1907 (3)	0.11066 (14)	0.76588 (10)	0.0515 (3)	
H7A	0.1646	0.1949	0.7532	0.062*	
C8	0.3530 (3)	0.08465 (14)	0.73113 (10)	0.0543 (4)	
H8A	0.3797	0.0001	0.7420	0.065*	
C9	0.4934 (2)	0.18093 (14)	0.67652 (10)	0.0488 (3)	
C10	0.4416 (2)	0.31192 (13)	0.64783 (8)	0.0434 (3)	
C11	0.2682 (2)	0.30333 (14)	0.58322 (9)	0.0463 (3)	
C12	0.1198 (3)	0.16483 (16)	0.54764 (11)	0.0588 (4)	
H12A	0.0640	0.1744	0.4898	0.088*	
H12B	0.0030	0.1345	0.5824	0.088*	
H12C	0.1978	0.0973	0.5480	0.088*	
C13	0.3573 (2)	0.54308 (14)	0.58070 (8)	0.0447 (3)	
C14	0.3076 (3)	0.66120 (16)	0.54642 (10)	0.0547 (4)	
H14A	0.1939	0.6494	0.5037	0.066*	
C15	0.4265 (3)	0.79222 (17)	0.57609 (12)	0.0622 (4)	
H15A	0.3928	0.8693	0.5535	0.075*	
C16	0.5992 (3)	0.81211 (15)	0.64035 (12)	0.0596 (4)	
H16A	0.6782	0.9020	0.6602	0.072*	
C17	0.6522 (3)	0.70013 (15)	0.67399 (10)	0.0511 (3)	
H17A	0.7675	0.7145	0.7162	0.061*	
C18	0.5324 (2)	0.56215 (13)	0.64491 (8)	0.0422 (3)	
C19	0.5751 (2)	0.44052 (13)	0.67829 (8)	0.0414 (3)	
C20	0.7569 (2)	0.45328 (13)	0.74642 (8)	0.0437 (3)	
C21	0.9681 (3)	0.50871 (19)	0.72893 (11)	0.0599 (4)	
H21A	0.9977	0.5393	0.6744	0.072*	
C22	1.1357 (3)	0.5186 (2)	0.79260 (14)	0.0710 (5)	
H22A	1.2772	0.5567	0.7807	0.085*	
C23	1.0937 (3)	0.4725 (2)	0.87317 (12)	0.0673 (5)	
H23A	1.2066	0.4777	0.9153	0.081*	
C24	0.8847 (3)	0.41878 (18)	0.89139 (11)	0.0623 (4)	
H24A	0.8563	0.3886	0.9461	0.075*	
C25	0.7164 (2)	0.40945 (15)	0.82857 (9)	0.0512 (3)	
H25A	0.5753	0.3736	0.8414	0.061*	

### Atomic displacement parameters (Å^2^)

**Table:**

	*U*^11^	*U*^22^	*U*^33^	*U*^12^	*U*^13^	*U*^23^
Cl	0.0863 (4)	0.1060 (5)	0.0916 (4)	−0.0055 (3)	0.0264 (3)	0.0365 (3)
O	0.0731 (7)	0.0532 (6)	0.0893 (8)	0.0308 (6)	0.0285 (6)	0.0164 (6)
N	0.0481 (6)	0.0462 (6)	0.0503 (6)	0.0133 (5)	0.0028 (5)	0.0012 (5)
C1	0.0537 (8)	0.0416 (7)	0.0515 (7)	0.0073 (6)	0.0036 (6)	0.0039 (5)
C2	0.0624 (9)	0.0487 (8)	0.0697 (10)	0.0146 (7)	0.0107 (7)	0.0116 (7)
C3	0.0731 (11)	0.0506 (8)	0.0718 (10)	0.0093 (7)	0.0057 (8)	0.0178 (7)
C4	0.0599 (9)	0.0639 (9)	0.0542 (8)	−0.0025 (7)	0.0051 (7)	0.0109 (7)
C5	0.0627 (10)	0.0746 (11)	0.0719 (11)	0.0178 (8)	0.0168 (8)	0.0097 (8)
C6	0.0667 (10)	0.0536 (8)	0.0677 (9)	0.0189 (7)	0.0126 (7)	0.0111 (7)
C7	0.0600 (8)	0.0358 (6)	0.0576 (8)	0.0121 (6)	0.0061 (6)	0.0067 (5)
C8	0.0636 (9)	0.0368 (6)	0.0634 (8)	0.0146 (6)	0.0107 (7)	0.0104 (6)
C9	0.0551 (8)	0.0377 (6)	0.0540 (7)	0.0135 (6)	0.0082 (6)	0.0043 (5)
C10	0.0478 (7)	0.0374 (6)	0.0460 (6)	0.0120 (5)	0.0112 (5)	0.0043 (5)
C11	0.0475 (7)	0.0404 (6)	0.0497 (7)	0.0102 (5)	0.0079 (5)	−0.0003 (5)
C12	0.0581 (8)	0.0442 (7)	0.0668 (9)	0.0065 (6)	−0.0008 (7)	−0.0035 (6)
C13	0.0488 (7)	0.0419 (6)	0.0454 (6)	0.0152 (5)	0.0093 (5)	0.0047 (5)
C14	0.0580 (8)	0.0511 (8)	0.0578 (8)	0.0214 (6)	0.0024 (6)	0.0100 (6)
C15	0.0742 (10)	0.0447 (7)	0.0734 (10)	0.0255 (7)	0.0080 (8)	0.0132 (7)
C16	0.0712 (10)	0.0368 (7)	0.0684 (9)	0.0124 (6)	0.0065 (7)	0.0008 (6)
C17	0.0579 (8)	0.0413 (7)	0.0522 (7)	0.0121 (6)	0.0040 (6)	0.0016 (5)
C18	0.0492 (7)	0.0374 (6)	0.0411 (6)	0.0127 (5)	0.0099 (5)	0.0036 (5)
C19	0.0466 (6)	0.0390 (6)	0.0403 (6)	0.0134 (5)	0.0099 (5)	0.0039 (5)
C20	0.0491 (7)	0.0386 (6)	0.0454 (6)	0.0153 (5)	0.0068 (5)	0.0021 (5)
C21	0.0529 (8)	0.0722 (10)	0.0563 (8)	0.0185 (7)	0.0134 (6)	0.0028 (7)
C22	0.0461 (8)	0.0879 (13)	0.0803 (12)	0.0219 (8)	0.0073 (8)	−0.0080 (9)
C23	0.0642 (10)	0.0716 (10)	0.0683 (10)	0.0301 (8)	−0.0129 (8)	−0.0077 (8)
C24	0.0742 (10)	0.0602 (9)	0.0508 (8)	0.0198 (8)	−0.0021 (7)	0.0065 (6)
C25	0.0544 (8)	0.0476 (7)	0.0488 (7)	0.0108 (6)	0.0058 (6)	0.0060 (5)

### Geometric parameters (Å, °)

**Table:**

Cl—C4	1.7399 (17)	C12—H12C	0.9600
O—C9	1.2149 (19)	C13—C18	1.4149 (19)
N—C11	1.3147 (18)	C13—C14	1.4173 (19)
N—C13	1.3646 (18)	C14—C15	1.365 (2)
C1—C6	1.380 (2)	C14—H14A	0.9300
C1—C2	1.397 (2)	C15—C16	1.403 (2)
C1—C7	1.467 (2)	C15—H15A	0.9300
C2—C3	1.384 (2)	C16—C17	1.367 (2)
C2—H2A	0.9300	C16—H16A	0.9300
C3—C4	1.379 (3)	C17—C18	1.4196 (19)
C3—H3A	0.9300	C17—H17A	0.9300
C4—C5	1.381 (3)	C18—C19	1.4253 (17)
C5—C6	1.385 (2)	C19—C20	1.4929 (18)
C5—H5A	0.9300	C20—C21	1.385 (2)
C6—H6A	0.9300	C20—C25	1.390 (2)
C7—C8	1.326 (2)	C21—C22	1.388 (2)
C7—H7A	0.9300	C21—H21A	0.9300
C8—C9	1.471 (2)	C22—C23	1.375 (3)
C8—H8A	0.9300	C22—H22A	0.9300
C9—C10	1.5142 (18)	C23—C24	1.374 (3)
C10—C19	1.3770 (18)	C23—H23A	0.9300
C10—C11	1.4267 (19)	C24—C25	1.384 (2)
C11—C12	1.5053 (19)	C24—H24A	0.9300
C12—H12A	0.9600	C25—H25A	0.9300
C12—H12B	0.9600		
			
C11—N—C13	118.73 (12)	N—C13—C18	122.82 (12)
C6—C1—C2	118.20 (15)	N—C13—C14	117.62 (13)
C6—C1—C7	118.85 (14)	C18—C13—C14	119.55 (13)
C2—C1—C7	122.95 (14)	C15—C14—C13	120.05 (14)
C3—C2—C1	120.99 (16)	C15—C14—H14A	120.0
C3—C2—H2A	119.5	C13—C14—H14A	120.0
C1—C2—H2A	119.5	C14—C15—C16	120.79 (14)
C4—C3—C2	119.06 (16)	C14—C15—H15A	119.6
C4—C3—H3A	120.5	C16—C15—H15A	119.6
C2—C3—H3A	120.5	C17—C16—C15	120.43 (14)
C3—C4—C5	121.40 (16)	C17—C16—H16A	119.8
C3—C4—Cl	119.65 (14)	C15—C16—H16A	119.8
C5—C4—Cl	118.96 (15)	C16—C17—C18	120.51 (14)
C4—C5—C6	118.56 (17)	C16—C17—H17A	119.7
C4—C5—H5A	120.7	C18—C17—H17A	119.7
C6—C5—H5A	120.7	C13—C18—C17	118.66 (12)
C1—C6—C5	121.79 (16)	C13—C18—C19	117.70 (12)
C1—C6—H6A	119.1	C17—C18—C19	123.63 (13)
C5—C6—H6A	119.1	C10—C19—C18	118.40 (12)
C8—C7—C1	126.86 (13)	C10—C19—C20	121.16 (12)
C8—C7—H7A	116.6	C18—C19—C20	120.42 (11)
C1—C7—H7A	116.6	C21—C20—C25	118.93 (14)
C7—C8—C9	124.21 (13)	C21—C20—C19	120.71 (13)
C7—C8—H8A	117.9	C25—C20—C19	120.37 (13)
C9—C8—H8A	117.9	C20—C21—C22	120.14 (16)
O—C9—C8	120.17 (13)	C20—C21—H21A	119.9
O—C9—C10	119.62 (13)	C22—C21—H21A	119.9
C8—C9—C10	120.21 (12)	C23—C22—C21	120.37 (16)
C19—C10—C11	119.78 (12)	C23—C22—H22A	119.8
C19—C10—C9	119.63 (12)	C21—C22—H22A	119.8
C11—C10—C9	120.30 (12)	C24—C23—C22	119.90 (16)
N—C11—C10	122.56 (12)	C24—C23—H23A	120.1
N—C11—C12	116.00 (13)	C22—C23—H23A	120.1
C10—C11—C12	121.44 (13)	C23—C24—C25	120.18 (16)
C11—C12—H12A	109.5	C23—C24—H24A	119.9
C11—C12—H12B	109.5	C25—C24—H24A	119.9
H12A—C12—H12B	109.5	C24—C25—C20	120.47 (15)
C11—C12—H12C	109.5	C24—C25—H25A	119.8
H12A—C12—H12C	109.5	C20—C25—H25A	119.8
H12B—C12—H12C	109.5		
			
C6—C1—C2—C3	0.4 (3)	C13—C14—C15—C16	0.2 (3)
C7—C1—C2—C3	−179.28 (15)	C14—C15—C16—C17	0.4 (3)
C1—C2—C3—C4	−0.5 (3)	C15—C16—C17—C18	−0.5 (3)
C2—C3—C4—C5	0.1 (3)	N—C13—C18—C17	−177.84 (13)
C2—C3—C4—Cl	179.88 (13)	C14—C13—C18—C17	0.8 (2)
C3—C4—C5—C6	0.2 (3)	N—C13—C18—C19	0.69 (19)
Cl—C4—C5—C6	−179.55 (14)	C14—C13—C18—C19	179.30 (12)
C2—C1—C6—C5	−0.1 (3)	C16—C17—C18—C13	−0.1 (2)
C7—C1—C6—C5	179.63 (16)	C16—C17—C18—C19	−178.57 (13)
C4—C5—C6—C1	−0.2 (3)	C11—C10—C19—C18	1.17 (19)
C6—C1—C7—C8	−176.02 (16)	C9—C10—C19—C18	174.99 (11)
C2—C1—C7—C8	3.7 (3)	C11—C10—C19—C20	179.45 (11)
C1—C7—C8—C9	−178.63 (14)	C9—C10—C19—C20	−6.74 (19)
C7—C8—C9—O	172.12 (16)	C13—C18—C19—C10	−1.45 (18)
C7—C8—C9—C10	−7.4 (2)	C17—C18—C19—C10	177.00 (13)
O—C9—C10—C19	−67.03 (19)	C13—C18—C19—C20	−179.74 (11)
C8—C9—C10—C19	112.49 (15)	C17—C18—C19—C20	−1.3 (2)
O—C9—C10—C11	106.75 (17)	C10—C19—C20—C21	114.65 (16)
C8—C9—C10—C11	−73.73 (18)	C18—C19—C20—C21	−67.11 (18)
C13—N—C11—C10	−0.7 (2)	C10—C19—C20—C25	−65.29 (17)
C13—N—C11—C12	179.73 (13)	C18—C19—C20—C25	112.95 (15)
C19—C10—C11—N	−0.1 (2)	C25—C20—C21—C22	0.6 (2)
C9—C10—C11—N	−173.84 (13)	C19—C20—C21—C22	−179.32 (15)
C19—C10—C11—C12	179.44 (13)	C20—C21—C22—C23	0.6 (3)
C9—C10—C11—C12	5.7 (2)	C21—C22—C23—C24	−1.3 (3)
C11—N—C13—C18	0.4 (2)	C22—C23—C24—C25	0.8 (3)
C11—N—C13—C14	−178.23 (13)	C23—C24—C25—C20	0.5 (2)
N—C13—C14—C15	177.86 (15)	C21—C20—C25—C24	−1.2 (2)
C18—C13—C14—C15	−0.8 (2)	C19—C20—C25—C24	178.80 (13)
